# Aβ_1–40_-Induced Platelet Adhesion Is Ameliorated by Rosmarinic Acid through Inhibition of NADPH Oxidase/PKC-δ/Integrin α_IIb_β_3_ Signaling

**DOI:** 10.3390/antiox10111671

**Published:** 2021-10-23

**Authors:** Bo Kyung Lee, Hye Jin Jee, Yi-Sook Jung

**Affiliations:** 1 College of Pharmacy, Ajou University, Suwon 16499, Korea; pfiffer@ajou.ac.kr (B.K.L.); hjjee@ajou.ac.kr (H.J.J.); 2 Research Institute of Pharmaceutical Sciences and Technology, Ajou University, Suwon 16499, Korea; 3 KIURI Research Center, Ajou University School of Medicine, Suwon 16499, Korea

**Keywords:** platelet, rosmarinic acid (RA), β-amyloid (Aβ), NADPH oxidase, integrin α_IIb_β_3_

## Abstract

In platelets, oxidative stress reportedly increases platelet adhesion to vessels, thus promoting the vascular pathology of various neurodegenerative diseases, including Alzheimer’s disease (AD). Recently, it has been shown that β-amyloid (Aβ) can increase oxidative stress in platelets; however, the underlying mechanism remains elusive. In the present study, we aimed to elucidate the signaling pathway of platelet adhesion induced by Aβ_1–40_, the major form of circulating Aβ, through Western blotting, immunofluorescence confocal microscopy, and fluorescence-activated cell sorting analysis. Additionally, we examined whether rosmarinic acid (RA), a natural polyphenol antioxidant, can modulate these processes. Our results show that Aβ_1–40_-induced platelet adhesion is mediated through NADPH oxidase/ROS/PKC-δ/integrin α_IIb_β_3_ signaling, and these signaling pathways are significantly inhibited by RA. Collectively, these results suggest that RA may have beneficial effects on platelet-associated vascular pathology in AD.

## 1. Introduction

Accumulating evidence suggests a correlation between vascular pathology and Alzheimer’s disease (AD) [1,2]. In numerous neuropathological studies, more than one-third of AD patients are accompanied by cerebrovascular lesions [3], and patients with vascular dementia also exhibit the hallmarks of AD, such as β-amyloid (Aβ) plaques and neurofibrillary tangles (NFTs) [4]. Several previous reports have suggested that vascular lesions induce Aβ deposition at the site of vascular damage, and elevated levels of circulating Aβ in the blood promote vascular lesions [5]. Additionally, various vascular risk factors such as atherosclerosis and hypercholesterolemia have been shown to increase the risk of AD [6].

Platelets are key cells that play a critical role in vascular pathology via thrombogenic activity, as well as by adhering to damaged vessels [1,7,8]. Recent studies have indicated a possible role of platelets in the pathology of vascular dementia and AD, given that platelets contain amyloid precursor proteins (APP) and α-, β-, and γ-secretases, which contribute to the production of circulating Aβ [9,10]. Abnormal platelet activity has also been reported in patients with AD [1]. In animal studies, Aβ injection enhanced platelet adhesion to injured blood vessels in a mouse model of carotid artery injury [5]. In platelets, reactive oxygen species (ROS), such as H_2_O_2_ and O_2_^−^, regulate platelet functions such as platelet aggregation and adhesion [11,12]. Therefore, various antioxidants afford preventive effects on platelet activation and thrombosis [13,14,15,16]. Furthermore, several recent studies have reported that oxidative stress occurs early in the brain of patients with AD [17], and Aβ increases ROS levels in platelets, resulting in platelet aggregation [11]. Collectively, these results suggest that Aβ-induced ROS in platelets may play a role in the vascular pathology of AD [18,19].

Rosmarinic acid (RA) is one of the major compounds commonly found in species of the family Boraginaceae and the subfamily Nepetoideae (Lamiaceae), and it is known to exhibit various biological activities, including antioxidative and anti-inflammatory effects [20,21]. Previous studies have reported that RA has antioxidant and antiaggregating activities in platelets [22]. Additionally, RA has shown efficacy in vascular diseases, such as diabetes and hypertension, mediated via its antiplatelet activity [22,23]. However, there is little information about the effect of RA on Aβ-induced platelet activation. In the present study, we investigated the effect of RA on Aβ-induced platelet adhesion and integrin α_IIb_β_3_, a major adhesion molecule in platelets. We also investigated the underlying mechanism of RA in terms of nicotinamide adenine dinucleotide phosphate (NADPH) oxidase and protein kinase C (PKC), which are major signaling molecules involved in integrin activation in platelets.

## 2. Materials and Methods

### 2.1. Reagents

RA was purchased from Sigma-Aldrich Co. (St. Louis, MO, USA), and Trolox was purchased from Tocris Bioscience (Ellisville, MO, USA). The following commercial antibodies were used: PKC-α, PKC-βI, PKC-βII, PKC-γ, PKC-δ (Santa Cruz Biotechnology, CA, USA), and β-actin (Cell Signaling Technology, Inc., Danvers, MA, USA). VAS2870 was purchased from Enzo Life Sciences (Farmingdale, NY, USA). Gö6976, rottlerin, and lucigenin were purchased from Sigma-Aldrich. Eptifibatide was purchased from Millipore (Burlington, MA, USA). CellTracker^TM^ Green 5-chloromethylfluorescein diacetate (CMFDA), 2,7-dichlorodihydro fluorescent diacetate (H_2_DCFDA), and dihydroethidium (DHE) were purchased from Thermo Fisher Scientific (Waltham, MA, USA). Fluorescein isothiocyanate (FITC) anti-human CD41a (integrin α_IIb_) antibody and APC anti-human CD61 (integrin β_3_) antibodies were purchased from BD Pharmingen (San Diego, CA, USA). All other chemical reagents were purchased from Sigma-Aldrich and were of analytical or high-performance liquid chromatography (HPLC) grade.

### 2.2. Washed Platelet Preparation

Freshly collected platelet-rich plasma (PRP) samples from healthy volunteers were procured from the Korean Red Cross Center, a blood donation facility for research purposes. The study was conducted in accordance with the Declaration of Helsinki, and the protocol was approved by the Ethics Committee of Ajou University (Project No. 202002-HM-EX-001). PRP was centrifuged at 1000× *g* for 10 min at 22 °C without breaking platelet pellets. The supernatant obtained was termed platelet-poor plasma. The obtained platelet pellet was washed twice with Tyrode’s buffer (pH 7.4).

### 2.3. Aβ_1–40_ Preparation

Briefly, Aβ protein fragment 1–40 (Aβ_1–40_; Abcam, San Francisco, CA, USA) peptides were dissolved in ammonium hydroxide (NH_4_OH; 4% final volume, Sigma-Aldrich) and then mixed with phosphate-buffered saline (PBS; pH 7.4, Invitrogen, Carlsbad, CA, USA) to obtain a 1 mg/mL solution. Aliquots were immediately stored at −80 °C and centrifuged for 15 min at 17,000× *g* prior to use to remove pre-aggregated materials.

### 2.4. Platelet Adhesion to Fibronectin

To observe platelet adhesion, stimulated platelets were stained with 10 μM CMFDA for 30 min at 37 °C. After washing with PBS, stained platelets (5 × 10^6^ cells/mL) were added to a glass-bottom dish coated with fibronectin (Sigma-Aldrich, St. Louis, MO, USA). After incubation for 1 h, nonadherent platelets were removed by washing with PBS. Platelets were captured in five fields per dish using a confocal microscope (Nikon, Japan). Adherent cells were calculated by expressing the areas of adherent platelets as a percentage of the total area. This experiment was repeated a total of three or five times.

### 2.5. Measurement of Filopodia Length and Spread Area in Platelet

For the measurement of the filopodial length and spread area of platelets, a glass-bottom dish was coated with fibronectin (Sigma-Aldrich, St. Louis, MO, USA). After washing with PBS, treated platelets (5 × 10^6^ cells/mL) were added to a glass-bottom dish. After incubation for 1 h, the image of live cells was acquired through differential interference contrast (DIC) imaging using a confocal microscope at 400–1000× magnification (Nikon, Japan). Filopodia, membrane protrusions supported by bundles, were randomly selected per each platelet. The filopodia length and spread area (excluding filopodia) were measured from 20 randomly selected fields with ImageJ software. This experiment was repeated a total four times.

### 2.6. Surface Expression of Integrins α_IIb_ and β_3_

In brief, the washed human platelets (2 × 10^8^ cells) were activated with 10 µM Aβ_1–40_ for 1 h at room temperature and then washed twice with Tyrode’s buffer and HEPES buffer. Next, the cells were immediately fixed with 2% paraformaldehyde on ice for 10 min. The cells were then washed twice in 1 × PBS, followed by the addition of 5 µL of FITC anti-human CD41a (integrin *α_IIb_*) or 5 µL of APC anti-human CD61 (integrin β_3_) to each sample and incubation for 30 min at room temperature in the dark. Finally, the cells were washed with 1 × PBS, resuspended in FACS sheath fluid, and analyzed using an FACSAria III flow cytometer (BD Biosciences, San Jose, CA, USA). This experiment was repeated a total of seven times.

### 2.7. Measurement of Free-Radical-Scavenging Activity by DPPH Assay

The free-radical-scavenging activity of RA was determined in vitro using the 2,2-diphenyl-1-picrylhydrazyl (DPPH; Sigma-Aldrich, St. Louis, MO, USA) assay as previously described. The protocol to assess DPPH radical scavenging activity was adapted from Brand-Williams et al. (1995), with minor changes [24]. Briefly, 100 μL aliquots of methanolic solutions containing different RA concentrations (0.1–30 μM) were added to 100 μL of a 250 μM methanolic DPPH solution. After 30 min, the absorbance was measured at 517 nm, and the percentage inhibition activity was calculated. The percentage (%) of DPPH free radical scavenging was calculated using the formula (A0 − A1)/A0 × 100, where A0 is the absorbance of the control, and A1 is the absorbance of the extract/standard. This experiment was repeated a total of three times.

### 2.8. ABTS Assay for Free-Radical-Scavenging Activity

The preformed radical monocation of 2,2’-azinobis(3-ethylbenzothiazoline-6-sulfonic acid (ABTS; Sigma-Aldrich, St. Louis, MO, USA) was generated as previously described [25]. The ABTS salt was weighed (19.3 mg) and dissolved in distilled water (5 mL). Then, 88 μL of K_2_S_2_O_8_ solution (0.0378 g/mL) was added to the ABTS solution, and the mixture was left at room temperature for 12 h in the dark. To perform measurements, the ABTS solution was diluted with ethanol to an absorbance of 0.700 ± 0.05 at 734 nm. Subsequently, 270 μL of the free-radical solution was combined with 20 μL of RA. The absorbance was measured at 734 nm after incubation at room temperature for 30 min in the dark. Antioxidant activity, expressed as a percentage of inhibition, was calculated using a previously established equation. Additionally, the half-maximal inhibitory concentration (IC_50_) was determined. This experiment was repeated a total of three times.

### 2.9. ROS Measurement

Briefly, washed human platelets (1 × 10^7^ cells), pretreated with RA, Trolox, or VAS 2870 for 30 min were incubated with either Aβ_1–40_ for 15 min, followed by incubation with 10 µM H_2_DCFDA or 10 µM DHE for 30 min. Fluorescence was immediately measured using an FACSAria III flow cytometer (BD Biosciences, San Jose, CA, USA) with an excitation wavelength of 488 nm and an emission wavelength of 530 nm. Gated cells (*n* = 10,000) were analyzed for each sample. This experiment was repeated a total of six times.

### 2.10. Measurement of NADPH Oxidase Activity

The activity of NADPH oxidase was determined in membrane fractions (50 μg of protein) incubated with 1 mM EGTA and 5 μM lucigenin in phosphate buffer (pH 7.0). The assay was initiated by adding 50 μM NADPH to the incubation mixture. Samples were immediately counted using a tabletop luminometer (Berthold Detection Systems FB Luminometer; Zylux Corp., Oak Ridge, Tennessee), with sampling performed every 6 s over a 5 min period; the fluorescence values were recorded for over 2 min of stable readings and averaged for each sample. This experiment was repeated a total of seven times.

### 2.11. Preparation of the PKC Membrane Fraction

Platelets were lysed using different lysis buffers, and lysates were extracted using different procedures. Briefly, the cells were incubated in lysis buffer A (20 mM Tris-HCl, pH 7.4, 250 mM sucrose, 1 mM EDTA, 0.1 mM NaF, 0.2 mM Na_3_VO_4_, 0.5 mM phenylmethylsulphonyl fluoride, 0.01 mM leupeptin, and 0.01 mg/mL aprotinin) for 30 min on ice and then centrifuged at 200,000× *g* in a Beckman Optima TL Ultracentrifuge (Beckman Coulter, Brea, CA, USA) at 4 °C for 30 min. The supernatants (cytosolic fractions, CFs) were removed, and the remaining pellets were resuspended in lysis buffer B (lysis buffer A containing 1% Triton X-100) and incubated on ice for 1 h. The suspension was then centrifuged as described above, and the supernatant (membrane fraction, MF) was obtained. This experiment was repeated a total of three times.

### 2.12. Western Blot Analysis

Western blotting was performed by modifying a previously described procedure [26]. Briefly, platelets were lysed in buffer A and B and centrifuged at 20,000× *g* for 30 min, and the supernatant was collected to obtain the cell membrane lysate. Then, proteins were separated using sodium dodecyl sulfate polyacrylamide gel electrophoresis and reacted with anti-PKC-α antibody (1:500, Santa Cruz), anti-PKC-βI (1:500, Santa Cruz), anti-PKC-βII (1:500, Santa Cruz), anti-PKC-γ (1:500, Santa Cruz), anti-PKC-δ (1:500, Santa Cruz), or anti-actin (1:3000, Cell Signaling) overnight. All samples were analyzed using a LAS 4000 mini (Fuji Photo Film, Tokyo, Japan). This experiment was repeated a total of three times.

### 2.13. Statistical Analysis

All data are expressed as the mean ± standard error of mean (SEM). Two-tailed *t*-tests were performed to examine differences in continuous variables, overall and at each time point, investigated in different comparison groups. Differences were considered statistically significant at *p* < 0.05.

## 3. Results

### 3.1. RA Reduces Aβ_1–40_-Induced Platelet Adhesion via Integrin α_IIb_β_3_ Blockade

**Figure 1:**
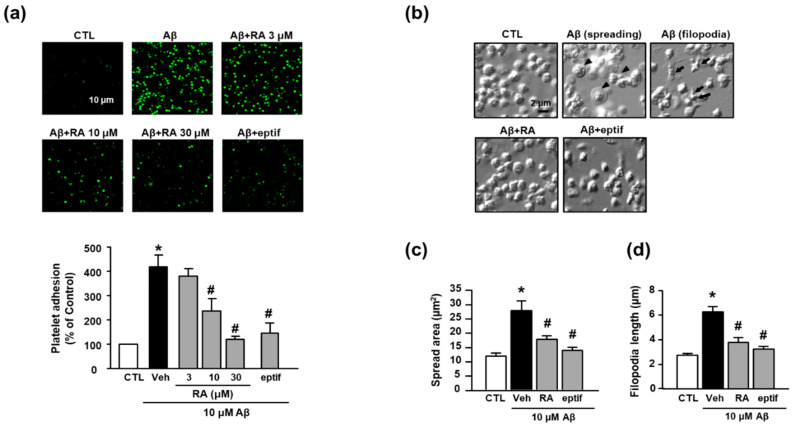
RA reduces Aβ_1–40_-induced platelet adhesion possibly through blockade of integrin α_IIb_β_3_. (**a**) Representative fluorescence image (upper) and quantitative analysis (bottom) of platelet adhesion to fibronectin. Platelet adhesion (green) was quantified following stimulation with 10 μM Aβ_1–40_ for 1 h, with or without RA (3, 10, and 30 μM) and 50 μM eptifibatide (eptif, an integrin α_IIb_β_3_ inhibitor) pretreatment for 30 min on fibronectin-coated coverslips. Data are presented as the mean ± SEM of five experiments. Scale bars represent 10 μm. (**b**) DIC images representative of platelet adhesion to fibronectin. Arrowheads and arrows indicate spreading and filopodia formation of platelets, respectively. Platelets were stimulated with 10 μM Aβ_1–40_ for 1 h, with or without 30 μM RA and 50 μM eptif pretreatment for 30 min. Scale bars represent 2 μm. (**c**) Quantitative analysis of the surface area of platelet spread; (**d**) quantitative analysis of filopodia length of platelets. Data are presented as the mean ± SEM in five or more random fields in three separate experiments. * *p* < 0.05 compared with CTL, # *p* < 0.05 compared with Veh. RA, rosmarinic acid; SEM, standard error of the mean; DIC, differential interference contrast; Veh, vehicle; CTL, control.

In the present study, we observed that Aβ_1–40_ increased platelet adhesion to fibronectin (418.65% ± 47.39%), and RA suppressed this increase in a concentration-dependent manner (236.57% ± 50.83% (10 μM) and 120.34% ± 12.62% (30 μM)) (Figure 1a). To determine whether adhesion to fibronectin is mediated by integrin α_IIb_β_3_, we examined the effect of eptifibatide, a specific integrin α_IIb_β_3_ inhibitor, on Aβ_1–40_-mediated platelet adhesion. Accordingly, eptifibatide significantly suppressed the Aβ_1–40_-mediated increase in platelets adhesion (147.21% ± 41.77%). Furthermore, Aβ_1–40_ promoted platelet spreading and filopodia to alter the platelet shape (Figure 1b). Aβ_1–40_ increased platelet spreading (Figure 1c) and filopodia length (Figure 1d) (27.74 ± 10.23 μm^2^ and 6.30 ± 1.05 μm, respectively); these changes were inhibited by RA (17.78 ± 3.35 μm^2^ and 3.78 ± 1.03 μm, respectively) and eptifibatide (13.82 ± 2.95 μm^2^ and 3.23 ± 0.55 μm, respectively).

**Figure 2:**
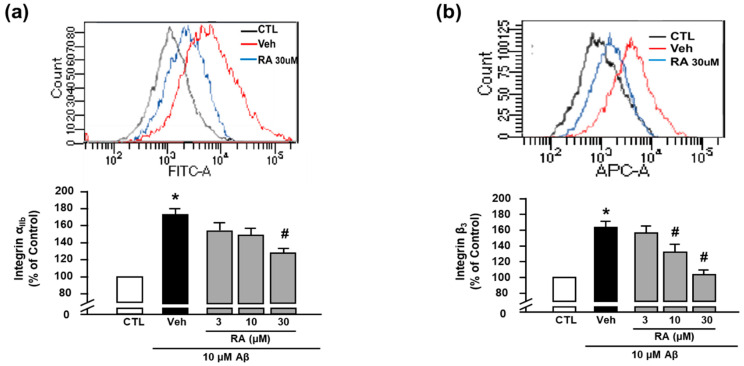
RA reduces Aβ_1–40_-induced activation of integrin α_IIb_β_3_. (**a**) Integrin α_IIb_ levels measured through flow cytometry. Representative flow cytometry histogram (upper) and quantitative analysis (bottom) of FITC–integrin α_IIb_-expressed platelets. (**b**) Integrin β_3_ levels measured through flow cytometry. Representative flow cytometry histogram (upper) and quantitative analysis (bottom) of APC–integrin β_3_-expressed platelets. Platelets were stimulated with 10 μM Aβ_1–40_ for 1 h with or without RA (3, 10, and 30 μM) pretreatment for 30 min. Data are presented as the mean ± SEM of seven experiments. * *p* < 0.05 compared with CTL, # *p* < 0.05 compared with Veh. FITC, fluorescein isothiocyanate. APC, allophycocyanin.

We next evaluated the activity of integrin α_IIb_ and integrin β_3_ using FITC anti-human CD41a antibody and APC anti-human CD61 antibody, respectively, through flow cytometry [8]. We observed that Aβ_1–40_ increased the activity of integrin α_IIb_ (176.73% ± 17.60%), and RA (30 μM) suppressed this increase (124.11% ± 12.86%) (Figure 2a). Additionally, Aβ_1–40_ increased the activity of integrin β_3_ (163.86% ± 7.08%), which was suppressed by RA (132.37% ± 9.66% at 10 μM and 104.05% ± 5.78% at 30 μM) (Figure 2b). These findings suggest that RA reduces Aβ_1–40_-induced platelet adhesion by blocking integrin α_IIb_β_3_. Since these results show only the activity of each integrin α_IIb_ and β_3_, further study is needed to clarify whether the clasping/unclasping mechanism is involved in the effects of RA on integrin α_IIb_β_3_.

### 3.2. Antioxidant Activity of RA

**Figure 3:**
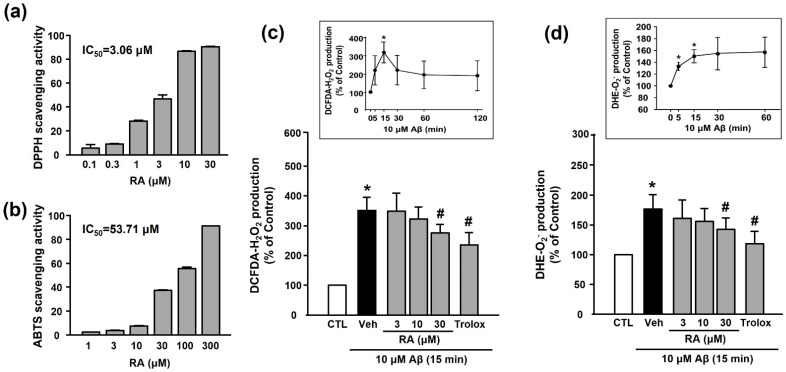
Antioxidant activity of RA in vitro and in platelets. (**a**) DPPH radical-scavenging activity of RA in vitro. Data are presented as the mean ± SEM of three individual experiments. (**b**) ABTS radical-scavenging activity of RA in vitro. Data are presented as the mean ± SEM of three individual experiments. (**c**) Aβ_1–40_-induced H_2_O_2_ production in platelets quantified by measuring DCF-DA fluorescence intensity. (c, insert) Time course of Aβ_1–40_-stimulated H_2_O_2_ generation. Platelets were treated with 10 µM Aβ_1–40_ for indicated time periods (0–120 min) in the presence of DCF-DA. Data are presented as the mean ± SEM of six experiments. (**d**) Aβ_1–40_-induced O_2_^−^ production in platelets quantified by measuring DHE fluorescence intensity. (**d**, insert) Time course of Aβ_1–40_-stimulated O_2_^−^ generation. Data are presented as the mean ± SEM of six experiments. * *p* < 0.05 compared with CTL, # *p* < 0.05 compared with Veh. DPPH, 2,2-diphenyl-1-picrylhydrazyl; DHE, dihydroethidium; ABTS, 2,2’-azinobis(3-ethylbenzothiazoline-6-sulfonic acid; DCF-DA, 2’-7’-dichlorodihydrofluorescein diacetate; ROS, reactive oxygen species.

We examined the in vitro antioxidant activity of RA using the DPPH and ABTS assays. As shown in Figure 3a,b, RA exhibited DPPH and ABTS radical-scavenging activities in a concentration-dependent manner. As shown in Figure 3c,d, Aβ_1–40_ increased ROS (H_2_O_2_ or O_2_^−^) production (351.50% ± 43.38% or 176.81% ± 24.37%, respectively), and this increase was inhibited by 30 μM RA (275.14% ± 29.61% or 142.56% ± 19.71%, respectively) and 100 μM Trolox (240.03% ± 40.99% or 118.70% ± 20.84%, respectively).

### 3.3. Effects of Trolox on Aβ_1–40_-Induced Platelet Adhesion and Integrin α_IIb_β_3_ Activation

**Figure 4:**
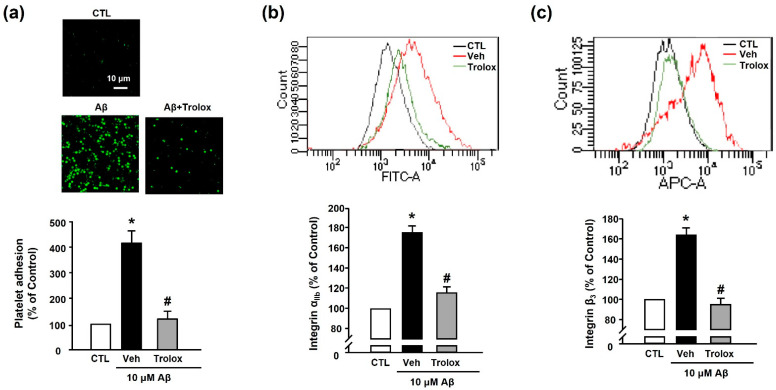
Effects of Trolox on Aβ_1–40_-induced platelet adhesion and integrin α_IIb_β_3_ activation. (**a**) Representative fluorescence image (upper) and quantitative analysis (bottom) of platelet adhesion to fibronectin. Adhesion of platelets (green) was quantified following stimulation with 10 μM Aβ_1–40_ for 1 h, with or without 100 μM Trolox pretreatment for 30 min on fibronectin-coated coverslips. Data are presented as the mean ± SEM of five experiments. Scale bars represent 10 μm. (**b**) Integrin α_IIb_ levels measured using flow cytometry. Representative flow cytometry histogram (upper) and quantitative analysis (bottom) of FITC–integrin α_IIb_-expressed platelets. (**c**) Integrin β_3_ levels measured using flow cytometry. Representative flow cytometry histogram (upper) and quantitative analysis (bottom) of APC–integrin β_3_-expressed platelets. Platelets were stimulated with 10 μM Aβ_1–40_ for 1 h, with or without 100 μM Trolox pretreatment for 30 min. Data are presented as the mean ± SEM of seven experiments. * *p* < 0.05 compared with CTL, # *p* < 0.05 compared with Veh.

Next, we used Trolox to examine whether Aβ_1–40_-induced platelet adhesion was associated with oxidative stress. We observed that 100 μM Trolox almost completely inhibited the Aβ_1–40_-induced increase in platelet adhesion (119.98% ± 30.71%) (Figure 4a). Additionally, Aβ_1–40_-induced integrin α_IIb_ and integrin β_3_ activation (176.73% ± 17.60% and 163.86% ± 7.08%, respectively) was suppressed following treatment with Trolox (117.89% ± 2.53% and 94.83% ± 6.32%, respectively) (Figure 4b,c).

### 3.4. RA Decreases Aβ_1–40_-Induced Platelet Activation Possibly through Inhibition of NADPH Oxidase

**Figure 5:**
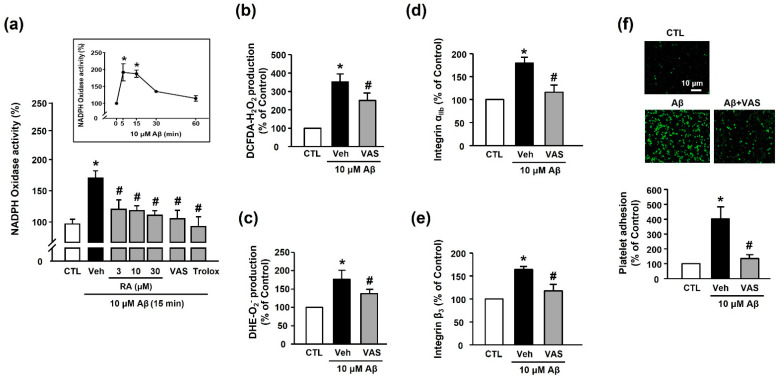
RA inhibits Aβ-induced platelet activation possibly through inhibition of NADPH oxidase. (**a**) Effect of RA on NADPH oxidase activity in platelets. Platelets were pretreated with RA (3, 10, and 30 μM), 10 μM VAS2870, or 100 μM Trolox for 30 min, followed by 15 min incubation with or without Aβ_1–40_. (insert) Time course of NADPH oxidase activity following platelet stimulation by Aβ_1–40_. Platelets were treated with 10 μM Aβ_1–40_ for the indicated time periods (0–60 min). Data are presented as the mean ± SEM of six experiments. (**b**) Aβ_1–40_-induced H_2_O_2_ production in platelets. (**c**) Aβ_1–40_-induced O_2_^−^ production in platelets. O_2_^−^ and O_2_^−^ production were measured by DCF-DA and DHE fluorescence intensity, respectively. Platelets were stimulated with 10 μM Aβ_1–40_ for 15 min with or without 10 μM VAS2870 pretreatment for 30 min. Data are presented as the mean ± SEM of three experiments. (**d**) Quantitative analysis of FITC–integrin α_IIb_-expressed platelets. (**e**) Quantitative analysis of APC–integrin β_3_-expressed platelets. Platelets were stimulated with 10 μM Aβ_1–40_ for 1 h with or without 10 μM VAS2870 pretreatment for 30 min. Data are presented as the mean ± SEM of seven experiments. (**f**) Representative fluorescence image (upper) and quantitative analysis (bottom) of platelet adhesion to fibronectin. Adhesion of platelets (green) was quantified following stimulation with 10 μM Aβ_1–40_ for 1 h with or without 10 μM VAS2870 pretreatment for 30 min on fibronectin-coated coverslips. Data are presented as the mean ± SEM of three experiments. * *p* < 0.05 compared with CTL, # *p* < 0.05 compared with Veh.

We measured the NADPH oxidase activity to determine the source of Aβ_1–40_-induced ROS generation. Our results revealed that Aβ_1–40_ increased NADPH oxidase activity (173.48% ± 11.52%) in platelets; this effect was suppressed by treatment with RA (3, 10, and 30 μM), 10 μM VAS2870 (NADPH oxidase inhibitor), and 100 μM Trolox (114.16% ± 6.83% (30 μM RA), 108.76% ± 16.09% (VAS2870), and 108.59% ± 13.01% (100 μM Trolox)) (Figure 5a). Additionally, ROS production, including H_2_O_2_ or O_2_^−^, was reduced following the suppression of NADPH oxidase activity (250.41% ± 40.88% or 142.57% ± 19.72%, respectively, Figure 5b,c). This finding suggests that the Aβ_1–40_-mediated increase in ROS levels was induced via the activation of NADPH oxidase. As shown in Figure 5d,e, the Aβ_1–40_-mediated increase in integrin α_IIb_ and integrin β_3_ activity (176.7%3 ± 17.60% and 163.86% ± 7.08%, respectively) was significantly inhibited by NADPH oxidase inhibition (117.65% ± 6.89% and 117.61% ± 14.44%, respectively). Additionally, the NADPH oxidase inhibitor completely suppressed Aβ_1–40_-induced platelet adhesion (128.23% ± 15.46%) (Figure 5f). These findings suggested that integrin α_IIb_β_3_ activity plays an important role in mediating platelet adhesion via NADPH oxidase, and RA could regulate this process.

### 3.5. RA Decreases Aβ_1–40_-Induced Platelet Activation Possibly through Inhibition of PKC-δ

**Figure 6:**
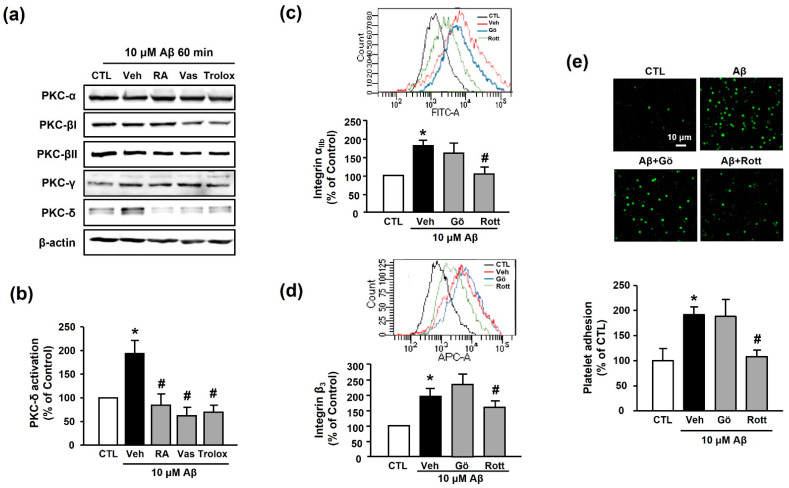
RA inhibits Aβ-induced platelet activation possibly through inhibition of PKC-δ. (**a**) Western blot analysis of PKC isotype (-α, -βI, -βII, -γ, and -δ) levels in Aβ_1–40_-stimulated platelets. (**b**) Quantitative analysis of PKC-δ level in Aβ_1–40_-stimulated platelets using Western blot analysis. Platelets were treated with 10 μM Aβ_1–40_ for 1 h with or without 30 μM RA, 10 μM VAS2870, or 100 μM Trolox pretreatment for 30 min. Data are presented as the mean ± SEM of three experiments. (**c**) Representative flow cytometry histogram (insert) and quantitative analysis of FITC–integrin α_IIb_-expressed platelets. (**d**) Representative flow cytometry histogram (insert) and quantitative analysis of APC–integrin β_3_-expressed platelets. Platelets were stimulated with 10 μM Aβ_1–40_ for 1 h with or without 1 μM classical PKC inhibitor (Gö6976) or 1 μM PKC-δ inhibitor (rottlerin) pretreatment for 30 min. Data are presented as the mean ± SEM of seven experiments. (**e**) Representative fluorescence image (upper) and quantitative analysis (bottom) of platelet adhesion to fibronectin. Adhesion of platelets (green) was quantified following stimulation with 10 μM Aβ_1–40_ for 1 h with or without 1 μM Gö6976 or 1 μM rottlerin pretreatment for 30 min on fibronectin-coated coverslips. Data are presented as the mean ± SEM of three experiments. * *p* < 0.05 compared with CTL, # *p* < 0.05 compared with Veh. PKC, protein kinase C.

The PKC family is an essential signaling mediator for platelet activation and aggregation [27]. Accordingly, we examined the potential role of the PKC family in RA-mediated suppression of platelet adhesion induced by Aβ_1–40_. Among the various PKC families, Aβ_1–40_ only increased the expression of PKC-δ (194.11% ± 27.24%); the increased PKC-δ activity was inhibited by 30 μM RA, 10 μM VAS2870, and 100 μM Trolox pretreatment (84.44% ± 23.62%, 62.02% ± 18.16%, and 69.78% ± 14.65%, respectively) (Figure 6a,b). Additionally, the Aβ_1–40_-mediated increases in integrin α_IIb_ and integrin β_3_ levels (176.73% ± 17.60% and 194.41% ± 26.33%) were significantly decreased by the PKC-δ inhibitor, 1 μM rottlerin (116.79% ± 6.14% and 159.41% ± 21.42%, respectively), but not by the classical PKC inhibitor, 1 μM Gö6976 (167.41% ± 22.57% and 234.93% ± 31.61%, respectively) (Figure 6c,d). Similarly, the Aβ_1–40_-induced increase in platelet adhesion (191.94% ± 15.42%) was blocked only by rottlerin (108.55% ± 12.25%) (Figure 6e). These results indicate that inhibitory effect of RA on Aβ_1–40_-induced platelet adhesion may be mediated through inhibition of not only NADPH oxidase but also PKC-δ.

## 4. Discussion

In the present study, we for the first time demonstrated the involvement of NADPH oxidase/ROS/PKC-δ/integrin α_IIb_β_3_ signaling in the mechanism of Aβ_1–40_-induced platelet adhesion. We further revealed that Aβ_1–40_-induced platelet adhesion is ameliorated by RA through inhibition of these signaling pathways.

The heterogeneous cleavage pattern of APP by β- and γ-secretase results in the production of Aβ peptides of varying lengths [28]. Reportedly, Aβ_1–40_ is the primary blood form of Aβ, contributing to vascular amyloid deposition in AD; Aβ_1–42_ is the predominant form in neural plaques [29]. Aβ_1–40_ accounts for more than 90% of Aβ produced from APP in the body and is the predominant type released from activated human platelets. Although Aβ_1–42_ has been found to mediate platelet aggregation, the efficacy of Aβ_1–42_ on vascular events is suggested to be far less than that mediated by Aβ_1–40_ [30,31]. Despite numerous investigations examining the correlation between AD and vascular lesions, studies on Aβ_1–40_ are relatively scarce when compared with those on Aβ_1–42_. Previously, we reported that the altered miRNA profile in platelets from patients with Alzheimer’s pathology was similar to that of Aβ_1–40_-exposed platelets in vitro [32], suggesting that Aβ_1–40_-stimulated platelets could play an important role during the process of Alzheimer’s pathology. Consistent with our previous report, the present study revealed that Aβ_1–40_ increased integrin α_IIb_β_3_ levels in human platelets, and that platelet adhesion to fibronectin was almost completely suppressed by a specific integrin α_IIb_β_3_ inhibitor. Additionally, RA suppressed platelet adhesion and integrin α_IIb_β_3_ activity in a concentration-dependent manner (Figure 1 and Figure 2), indicating that the effect of RA on Aβ_1–40_-induced platelet adhesion may be mediated via integrin α_IIb_β_3_.

Numerous studies have reported that oxidative stress contributes to Aβ generation and NFT formation, suggesting a close association between amyloid plaques and ROS in the pathogenesis of AD [19,33,34]. Elevated ROS production reportedly increases the activity of β- and γ-secretases, which leads to increased APP cleavage and Aβ generation. Furthermore, in patients with AD, lipid peroxidation markers, such as 4-hydroxynonenal and malondialdehyde, were found to be elevated in the peripheral tissues, possibly because of insufficient enzymatic/nonenzymatic antioxidants [35,36]. The present study revealed that Aβ_1–40_ significantly increased platelet ROS levels, and the levels of O_2_^−^ or H_2_O_2_ began to increase rapidly at 5 min, peaking at 15 min after Aβ_1–40_ exposure (Figure 3). RA significantly reduced elevated ROS levels in a concentration-dependent manner. Our results further revealed that Trolox, a powerful antioxidant, significantly inhibited both the Aβ_1–40_-induced increase in platelet adhesion and the integrin α_IIb_β_3_ activation. These results suggest that Aβ_1–40_-induced ROS may activate platelet adhesion, consistent with the findings of a recent study indicating a potential role for platelets in the pathogenesis of AD [2,11,37]. Our results further indicate that the inhibitory effect of RA on Aβ_1–40_-induced platelet adhesion may occur through its antioxidant activity.

Enzyme pathways known to induce ROS generation include NADPH oxidase, myeloperoxidase, xanthine oxidase, or uncoupled nitric oxide synthase. In particular, NADPH oxidase, an enzyme complex composed of several subunits and a small GTPase Rac, has been reported to play a role in some neurodegenerative diseases, including dementia, via ROS-induced neuronal death [38,39]. Additionally, NADPH oxidase is suggested to be a major source of Aβ-induced ROS in hippocampal cells [38,40]. Furthermore, growing evidence suggests that the enzymatic activity of NADPH oxidases plays a significant role in promoting platelet function [11,16]. Among the seven NADPH oxidase isotypes, only NADPH oxidases 1/2 are expressed in human platelets and are closely related to platelet activity. However, the molecular mechanism underlying Aβ_1–40_-induced platelet activation remains poorly understood [14,41]. According to our findings, Aβ_1–40_ increased NADPH oxidase activity, and the NADPH oxidase inhibitor VAS2870 inhibited Aβ_1–40_-induced platelet adhesion and integrin α_IIb_β_3_ activity. These results indicate that NADPH oxidase plays an important role in Aβ_1–40_-induced platelet activity, especially in terms of integrin α_IIb_β_3_ activity (Figure 5). Although Aβ_1–40_-induced NADPH oxidase isotype-specific activity was not detected in this study, it is speculated that Aβ_1–40_ activated NADPH oxidases 1/2, as they are the primary forms present in platelets. In the present study, the Aβ_1–40_-induced increase in H_2_O_2_ and O_2_^−^ levels was blocked by inhibiting NADPH oxidase activity and vice versa. Alternatively, Trolox decreased NADPH oxidase activity, probably owing to the rapid scavenging of generated O_2_^−^ following Trolox pretreatment, as the NADPH oxidase activity was measured by detecting O_2_^−^ using lucigenin.

Various cascades regulate integrin α_IIb_β_3_ levels in platelets, among which PKC is a well-known factor [42]. However, the crosstalk between Aβ_1–40_ and PKC isotypes in platelets has not been previously elucidated. In the present study, we, for the first time, reported that, among several isotypes of PKC (-α, -βI, -βII, and -γ), the level of PKC-δ was remarkably increased by Aβ_1–40_. Additionally, Aβ_1–40_-induced PKC-δ activation was suppressed by RA, Trolox, or VAS2870 (Figure 6). Furthermore, we observed that rottlerin, a PKC-δ specific inhibitor, significantly inhibited the Aβ_1–40_-induced increase in integrin α_IIb_β_3_ activity and platelet adhesion; however, Gö6976, a nonspecific inhibitor of PKC-α, -β, and-γ, did not demonstrate this effect. These results suggest that Aβ_1–40_-induced integrin α_IIb_β_3_ activity could be regulated by PKC-δ, which produces a representative G-protein-coupled receptor signaling cascade. PKC is divided into isotypes depending on whether calcium ions mediate their regulation. PKC-α, -β, and -γ are regulated by calcium ions, whereas PKC-δ acts independently of calcium ions. Thus, it is suggested that Aβ_1–40_-induced integrin α_IIb_β_3_ activity may be regulated independently of calcium ions.

Overall, the underlying mechanisms for the inhibitory effect of RA on Aβ_1–40_-induced platelet adhesion may involve inhibition of NADPH oxidase/ROS/PKC-δ/integrin α_IIb_β_3_ signaling. Although reports have previously indicated a relationship between NADPH oxidase and integrin α_IIb_β_3_ activity in platelets [7,41], this However, the present study is the first to reveal a link between NADPH oxidase/PKC-δ and Aβ_1–40_-induced integrin α_IIb_β_3_ in the process of platelet adhesion. Our study further showed that RA inhibits all these signaling pathways, consequently inhibiting platelet adhesion. These results indicate that integrin α_IIb_β_3_ and NADPH oxidase can be potential therapeutic targets for platelet-associated vascular pathology in AD. Unsurprisingly, however, the most serious side-effects commonly observed with integrin α_IIb_β_3_ antagonists include bleeding and thrombocytopenia. Therefore, more specific integrin inhibitors involved in the pathological mechanism are needed. In addition, NADPH oxidase may exhibit different roles in other cells, thus highlighting the need for platelet-specific NADPH oxidase inhibitors. As for RA, it has been reported to attenuate the pathological function of integrin α_IIb_β_3_, and possess antithrombotic effect against Aβ [21,43,44]. Therefore, RA could be developed as a therapeutic agent for platelet-associated vascular pathol-ogy in AD.

## Data Availability

All of the data is contained within the article.
